# Scalable Combinatorial Assembly of Synthetic DNA for Tracking Applications

**DOI:** 10.3390/ijms24032549

**Published:** 2023-01-29

**Authors:** Julius D. Stuart, Natalie R. Wickenkamp, Kaleb A. Davis, Camden Meyer, Rebekah C. Kading, Christopher D. Snow

**Affiliations:** 1 Department of Chemistry, Colorado State University, Fort Collins, CO 80523, USA; 2 Department of Microbiology, Immunology, and Pathology, Colorado State University, Fort Collins, CO 80523, USA; 3 Department of Chemical and Biological Engineering, Colorado State University, Fort Collins, CO 80523, USA

**Keywords:** synthetic DNA tags, synthetic DNA barcoding, DNA assembly, computational design, surveillance, combinatorial library, next-generation sequencing

## Abstract

Synthetic DNA barcodes are double-stranded DNA molecules designed to carry recoverable information, information that can be used to represent and track objects and organisms. DNA barcodes offer robust, sensitive detection using standard amplification and sequencing techniques. While numerous research groups have promoted DNA as an information storage medium, less attention has been devoted to the design of economical, scalable DNA barcode libraries. Here, we present an alternative modular approach to sequence design. Barcode sequences were constructed from smaller, interchangeable blocks, allowing for the combinatorial assembly of numerous distinct tags. We demonstrated the design and construction of first-generation (N = 256) and second-generation (N = 512) modular barcode libraries, from fewer than 50 total single-stranded oligonucleotides for each library. To avoid contamination during experimental validation, a liquid-handling robot was employed for oligonucleotide mixing. Generating barcode sequences in-house reduces dependency upon external entities for unique tag generation, increasing flexibility in barcode generation and deployment. Next generation sequencing (NGS) detection of 256 different samples in parallel highlights the multiplexing afforded by the modular barcode design coupled with high-throughput sequencing. Deletion variant analysis of the first-generation library informed sequence design for enhancing barcode assembly specificity in the second-generation library.

## 1. Introduction

Inventory and internal stock management are common industrial practices for tracking the flow of raw materials and products from the point of manufacture to the point of sale [1]. Material and product tracking is executed, in part, through the application of a standard barcode. While barcodes, such as one-dimensional Universal Product Code (UPC) barcodes, remain an industry standard, these markers possess certain limitations capable of influencing a company’s security [2]. Specifically, UPC barcodes and two-dimensional Quick Response (QR) codes are *visible* markers rendering them susceptible to removal, subversive duplication, or counterfeiting. Moreover, the products must be large enough to display the barcodes thus limiting the types of products that can be tracked.

In contrast, molecular barcodes possess inherent characteristics surpassing the limitations of existing UPC barcodes. Molecular barcodes can be classified into two high-level categories: (1) non-sequence-encoding and (2) sequence-encoding [3]. Examples of non-sequence-encoding materials, as given by Paunescu et al., include fluorescent dyes and quantum dots that provide a unique optical signature serving as the barcode [3]. However, these materials are limited in the number of barcodes that can be generated due to spectral overlap. Sequence-encoding materials include synthetic polymers [4,5], peptides [6,7], and DNA [8,9]. These materials boast astronomically high numbers of possible barcodes, making them attractive as potential tracking tools. Of these, DNA stands out as the most accessible system due to the ongoing revolution in economical synthesis and sequencing [10].

For our purposes, synthetic DNA “barcodes” are short (~100–200 bp), double-stranded DNA duplexes of known sequence, intended to encode specific information relative to tracking biomaterials or organisms in natural systems. Our synthetically generated barcodes functionally serve as unique biomarkers for tagging and tracking purposes and are therefore not related to species identification barcoding [11] or the barcodes employed in NGS adaptors for multiplexing [12]. This size range is convenient for signal amplification via PCR, as well as reading the stored information via traditional or next-generation DNA sequencing (NGS). The feasibility of DNA serving as a tracking material has been tested in various applications [9,13]. Nanoparticle surface adsorbed DNA, in the form of silica-encapsulated DNA, has been studied as a tracking material for oils [14], trophic pathways [15], reservoir imaging [9], and aquifer characterization [8]. Surface adsorption is suggested to afford nucleic acid resistance to nucleases, an advantageous characteristic provided by the surface [16].

While sources in the literature have measured the stability of DNA-based barcodes [9], few sources have described practical scalable methods for DNA barcode sequence design and synthesis in the context of tracking applications. To be clear, many researchers have described novel encoding schemes for storing various information types (e.g., file content, books, digital media) in DNA [17,18,19]. Such encoding, while advantageous for information storage, falls beyond the purview of DNA barcodes as unique tracking tags, where scalable sequence generation and ease-of-detection and differentiation remain the primary focus.

This work describes a combinatorial DNA barcode sequence design strategy that allows the user to construct hundreds of unique sequence tags from fewer than 50 single-stranded oligonucleotides. To maximize fidelity and yield, the sequences were designed computationally using a custom Python code and the nucleic acid modeling and design software NUPACK [20]. Experimental validation of top-scoring candidate libraries was performed using NGS. The results herein demonstrate that modular DNA barcodes offer increased autonomy for the user, reducing oligonucleotide costs by taking advantage of combinatorial assembly. Combined with sensitive, amplification-mediated detection, modular barcodes offer an appealing alternative to conventional DNA-tagging methodologies that are cost-inefficient and rely heavily upon external nucleic acid synthesis.

## 2. Results

### 2.1. Modular Barcode Layout

**Figure 1:**
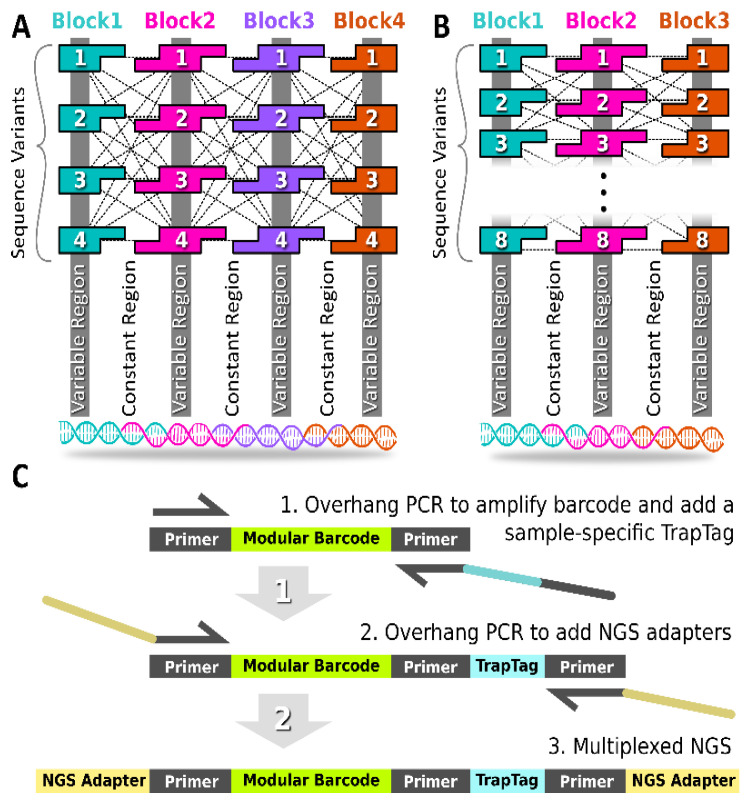
Modular Barcode Design. (**A**) The first-generation modular barcode library (gen_1) contained four blocks with single-stranded overhangs for mixing and annealing. For each block, four variants were obtained allowing the generation of 4^4^ (256) barcode sequences. (**B**) The second-generation modular barcode library (gen_2) contained three blocks. The eight variants per block allowed for generation of 8^3^ (512) sequences. (**C**) A UMI Trap Tag sequence was appended using overhang PCR to facilitate multiplex NGS sequencing of multiple samples in parallel.

The modular barcode was composed of multiple double-stranded DNA blocks in series (Figure 1A,B and Figure S1). The junction between neighboring blocks contained 10-base pair assembly-encoding single-stranded overhangs, such that single-stranded oligos annealed in the target order, requiring the overhang sequence domains to remain constant across all barcode variants (i.e., “constant regions”). The termini of each assembled barcode remained constant also, allowing the PCR amplification of any barcode variant in the library using the same primer set. The unique signature for each barcode resulted from regions internal to each block that are unique to each block variant (i.e., “variable regions”). The shared overhang domains allowed the combinatorial mixing of block variants to generate different barcode sequences. 

As shown in Table 1, the first-generation modular barcode library (Gen_1) was comprised of four blocks with each block harboring an internal 6-nucleotide (nt) variable sub-domain. Duplicate variable region sequences were permitted for the Gen_1 library. A total of four variants was obtained for all four blocks, allowing the construction of 256 (4^4^) unique sequences. The second-generation library (Gen_2) was comprised of three blocks with each block containing an internal 12 nt variable sub-domain. Duplicate variable region sequences were not permitted for the Gen_2 library. A total of eight block variants was obtained allowing the construction of 512 (8^3^) unique sequences. The reduced number of blocks in the Gen_2 library was motivated by off-target deletion variants observed in the Gen_1 library described below. Furthermore, the expanded variable sub-domain in the Gen_2 library (12 nt instead of 7 nt) allowed increased diversity (Hamming distance), thereby reducing the likelihood of barcode misidentification in the face of polymerase errors.

### 2.2. Trap Tag

For multiplexing in NGS, it is important to use unique molecular identifier (UMI) tags [21,22]. In the context of modular barcode detection, we refer to our UMI as a TrapTag, an 8-nucleotide sequence capable of representing the site and/or time of barcode DNA recovery (Figure 1C). Appending a TrapTag to a barcode amplicon allows one to track barcode DNA with the added dimension of location or time, thus increasing the resolution of tracking studies, while simultaneously increasing the information density of sequencing data. Critically, appending TrapTags increased the multiplexing capability of modular barcode libraries by allowing the simultaneous reading of multiple distinct collection samples via NGS. Without requiring additional equipment, TrapTags were appended using overhang PCR, a routine technique in modular barcode library preparation, thus preserving overall cost efficiency. The 100 TrapTag sequences purchased for this study were adopted from known Illumina sequencing adapters (Table S1).

### 2.3. Sequence Design and Specificity

Primer3 was used for designing barcode primer pairs (Table 2) for both Gen_1 and Gen_2 libraries (See SI, Primer design in Extended Methods). In contrast to that of Gen_1, the Gen_2 TrapTag primer sequence was designed using NUPACK, which considered off-target complex formations during sequence design for reducing potential off-target primer binding internal to barcode sequences. Laboratory evaluations of Gen_2 TrapTag primer specificity for barcode amplification in the context of insect (mosquito) or contaminating human DNA demonstrated clean amplification of the barcode target of the correct amplicon size (Figure S2). Amplification of barcode DNA was also highly sensitive, with successful amplification down to 10^−10^ of the initial barcode concentration (10 ng/µL) of the target sequence (Figure S2).

**Figure 2:**
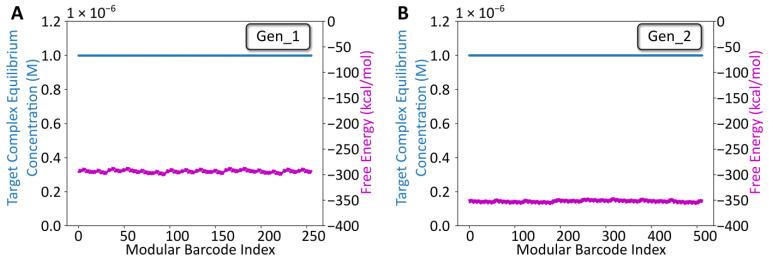
NUPACK Design Analysis. (**A**) Tube analysis results for the gen_1 library. The x-axis is the modular barcode index, a value assigned to each unique sequence within the library. The left y-axis corresponds to the target complex equilibrium concentration predicted using NUPACK after defining a tube containing the eight oligos at a starting concentration of 1 µM, at room temperature for each barcode. The right y-axis corresponds to the free energy for each barcode complex predicted using NUPACK. (**B**) Comparable Tube analysis results for the gen_2 library.

Following overhang sequence design using NUPACK, the sequences for both libraries (256 seqs for Gen_1, 512 seqs for Gen_2) were constructed in silico and analyzed using NUPACK’s Tube Analysis function (Figure 2). The NUPACK script is available on the Zenodo repository (DOI: 10.5281/zenodo.7415652). The analysis predicted the target complex equilibrium concentration and free energy, while considering off-target complexes (any competing arrangement of up to four strands). For both libraries, the target complex was predicted to dominate at equilibrium, and the free energy oscillated, minimally, around −300 kcal/mol (Gen_1) or −350 kcal/mol (Gen_2), suggesting that all combinatorial library members were likely to anneal as intended following mixing. Notably, the Gen_2 barcodes were longer (128 bp) than the Gen_1 barcodes (102 bp). Thus, the lower free energy estimate for Gen_2 library members reflects a comparable per-bp stability. The same primer design approach (Primer3 → Specificity check) was used for designing and analyzing the remaining TrapTag primer sequence for the Gen_2 library. Experimental validation was pursued following NUPACK analysis.

### 2.4. NGS Barcode Recovery

**Figure 3:**
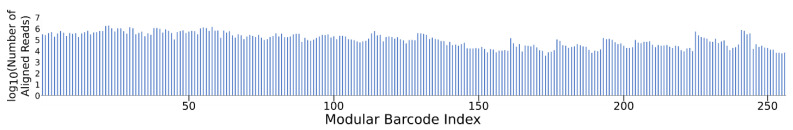
Gen_1 NGS recovery of all 256 modular barcodes. Here, ~80 M joined reads were aligned and assigned using a custom Python script. All barcodes were detected at read quantities ranging from 10^3^ to 10^6^, validating the multiplexing capability provided by the modular barcode design.

The NGS results for the pooled modular barcode library (Gen_1) confirmed the detection of all 256 sequences (within several orders of magnitude) within the entire dataset, in a single sequencing run (Figure 3). The wide read distribution likely resulted from inaccurate quantitation of individual barcodes prior to attempted equimolar mixing. NGS results for the Gen_2 library confirmed the recovery of all 96 pooled barcodes among ~110 M reads (Figure S4). These results highlight the multiplexing capability of the modular barcode design. Given the relatively low complexity of the barcode library, coupled with the intent to make data analysis manageable in terms of time and computational cost, the sequencing dataset was downsampled such that only 1 M reads were further analyzed out of the 76 M paired-end reads received for determining the number and type of deletion variants and 1 nt substitution variants. While downsampling, or subsampling as previously described [23], may not provide complete depiction of the results, any trends observed in the truncated dataset are likely to persist throughout the entire dataset, considering the high redundancy of the barcode NGS samples. It is likely that additional ‘block’ variants would allow parallel modular barcode detection to a greater degree than that demonstrated here with 256 barcodes constructed with only four variants for each of the four blocks. As shown in Table 3, out of the 1 M read subset for the Gen_1 library, 80% were perfectly aligned barcodes, 3% were deletion variants, and 5% were 1 nt substitution variants. For the 1 M read subset of the Gen_2 library, 26% were perfectly aligned barcodes, 1% were deletion variants, and 3% were 1 nt substitution variants. The remaining unclassified proportion of reads for Gen_1 (12%) and Gen_2 (70%) likely reflect a mixture of reads containing muti-nt substitutions and variable length insertions.

### 2.5. Deletion Analysis

**Figure 4:**
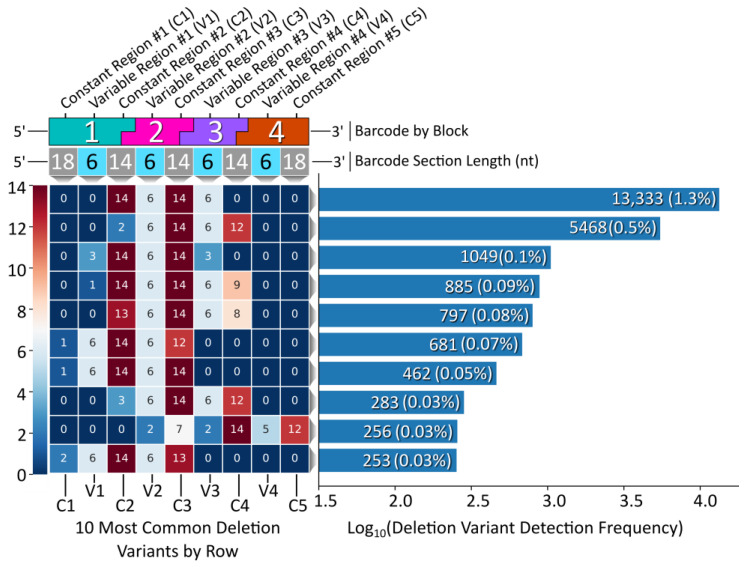
Gen_1 deletion variant analysis. Left, a heatmap displaying the top 10 deletion variants. Each block corresponds to a certain section within each barcode, top, with constant regions referring to overhang sequence domains between blocks and variable regions corresponding to the unique sequences internal to each block in the modular design. The number within each block represents the number of deleted nucleotides for that section. Right, a histogram displaying the detection frequency for the top 10 variants out of the 1 M read subset.

While the vast majority of the Gen_1 barcode amplicon reads consisted of full-length reads (80% of the 1 M read data sub-set had a length of 128 bp), the ample amount of data returned in the raw NGS sequencing results was sufficient to reveal relatively rare off-target barcodes. We therefore proceeded to assess the oligonucleotide assembly fidelity during annealing, the first step of modular barcode construction, and found amplicons indicative of off-target assembly events. Figure 4 displays a heatmap of the 10 most common deletion variants detected out of 1 M sequencing reads (Gen_1). The number of missing nucleotides within a given barcode segment ranged from 1 to 14, with the majority of deletion variants missing portions of constant regions 2 and 3 and variable regions 2 and 3 internal to blocks 2 and 3.

**Figure 5:**
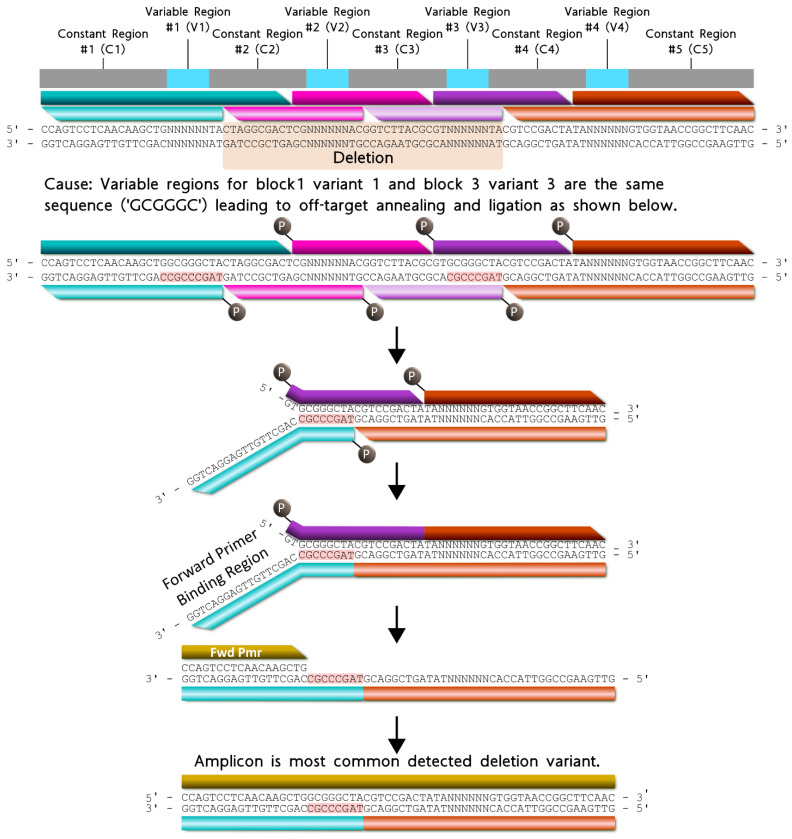
Proposed formation pathway for the highest deletion variant (gold) resulting from identical terminal nucleotides (red highlighted) for block 1 (turquoise) and block 3 (purple) bottom strands. All constant and variable regions across the modular barcode are represented by the colors gray and cyan, respectively. The deletion domain is indicated by the orange highlighted nucleotides. Blocks 2 and 4 are represented by the colors pink and orange respectively. For each block, the darker shade of each color represents the top strand, and the lighter shade of each color represents the bottom strand.

The most common deletion variant occurred more than twice as often as the second highest variant, suggesting that some aspect of the barcode design favored the formation of this off-target variant, warranting further inspection. Out of the 13,333 reads for the most common deletion variant, 13,306 contained the variable region sequence ‘GCGGGC’, the only duplicate variable region sequence used and shared by blocks 1 and 3, italicized in Table 4. Additionally, the 5′ terminal two nucleotides for the bottom strands of blocks 1 (turquoise) and 3 (purple) were identical. As shown in Figure 5, the shared sequence (variable region and terminus) allowed block 1 to anneal adjacent to block 4, becoming ligated. Such an off-target annealing event created a truncated oligo containing the barcode forward primer-binding region, in addition to the reverse primer sequence, allowing propagation throughout subsequent library preparation PCR stages. 

**Figure 6:**
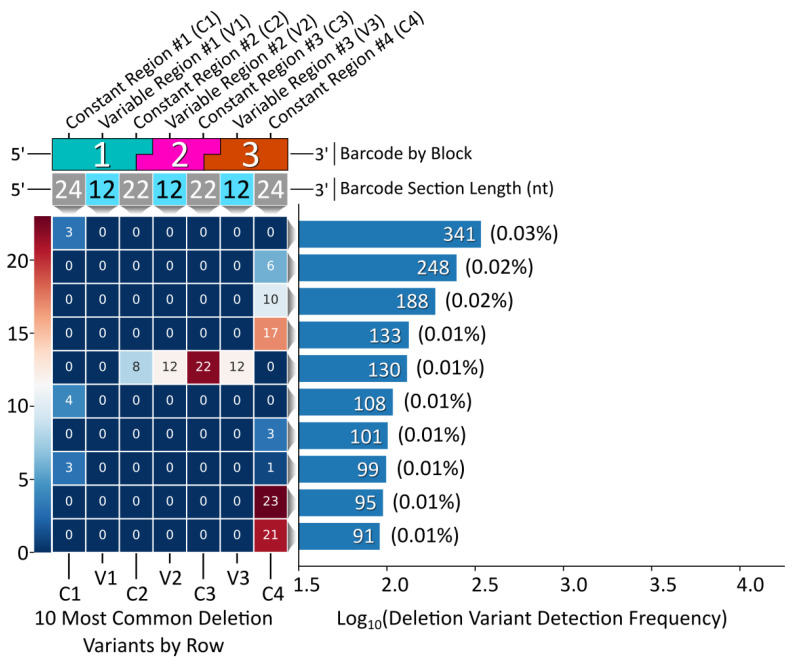
Gen_2 library deletion variant analysis. Left, a heatmap corresponding to the 10 most common deletion variants by row. Each block corresponds to a specific barcode domain displayed above the heatmap. Right, a histogram displaying the frequency of detection for each deletion variant out of the 1 M read subset.

Deletion variant analysis revealed a sequence motif to control for (i.e., negative design) during NUPACK sequence design. To avoid this problem in the subsequent Gen_2 modular barcode library, we specified a hard constraint for preventing duplicate nucleotides between block termini (Figure S3). Remarkably, the most common deletion variant from the pooled, 96 barcode Gen_2 library contained no internal deletions, rather a simple 3 nt terminal deletion (Figure 6). The fifth most common variant containing an internal deletion resulted from partial complementarity between blocks 1 and 3, suggesting an additional design principle for the design of subsequent libraries (Figure S5). Overall, Gen_2 library construction was improved by avoiding duplicate variable region sequences coupled with the NUPACK design constraint incorporation for eliminating duplicate 5′ terminal sequences adjacent to variable region sequences.

### 2.6. Substitution Analysis

**Figure 7:**
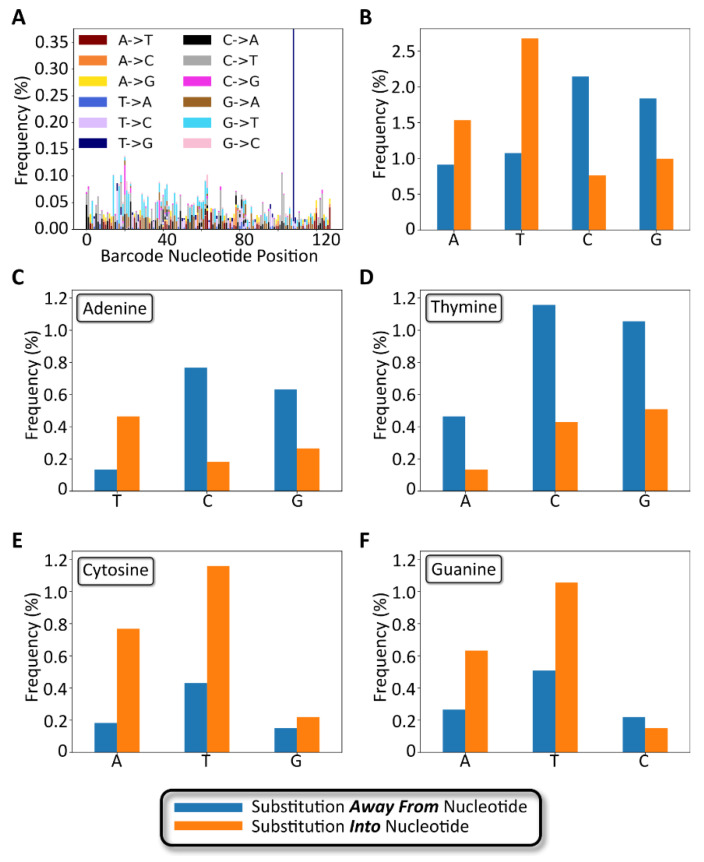
Gen_1 substitution variant analysis. (**A**) Stacked bar chart showing the number and type of 1-nucleotide substitutions plotted as a function of the nucleotide position along the barcode. (**B**) Bar chart showing nucleotide substitution frequency broken down by nucleotide. Blue bars represent substitutions away from the specified nucleotide. Orange bars represent substitutions to the specified nucleotide. (**C**–**F**) Bar charts for substitution frequency for each of the four bases, adenine, thymine, cytosine, and guanine, respectively.

The number and type of 1-nucleotide substitutions were analyzed to determine the extent to which a modular barcode library of low complexity relative to conventional sequencing datasets exhibits substitution errors and trends that coincide with previously published data. As shown in Figure 7A, the positions of 1 nt substitutions were distributed across the entire barcode sequence. The average per-nucleotide substitution frequency, as calculated among all full-length, non-insertion/deletion containing reads, was 0.05 ± 0.04% (Figure S7), slightly lower than a previously reported rate of 0.24 ± 0.06% [24]. The frequency of detected substitution types appeared to be random as well, with the exception of one substitution (T108G) that appeared with an overall frequency approximating 0.38%. We hypothesize that this particular substitution occurred early during library preparation PCR amplification of the barcode, rather than occurring as a random substitution during NGS.

The number and type of substitutions are broken down by nucleotide in Figure 7B–D. As shown in Figure 7B, substitutions away from the wild-type base occurred most frequently for cytosine, followed by guanine, with thymine and adenine exhibiting the fewest substitutions. However, the opposite trend was observed for substitutions into a specific base, with thymine and adenine occurring more frequently than guanine and cytosine. The most frequent substitution types were C -→ A, C -→ T, and G -→ T in decreasing order. The least frequent substitution types were A -→ C, C -→ G, G -→ C, and T -→ C in decreasing order. Notably, the above trends, with the exception of T -→ C as an infrequent substitution, matched trends previously reported by Pfeiffer et al., describing Illumina sequencing error rates (Table S2) [25]. The comparable substitution errors reported here with previously described data confirm that our low complexity modular barcode libraries did not suffer from elevated substitution errors that would frustrate sequencing data quality and accurate read identification.

## 3. Conclusions

Modular DNA barcode libraries represent an efficient combinatorial method of generating numerous, NGS-compatible synthetic DNA sequences for tracking applications. Rigorous computational design and scoring ensured that all single-stranded oligo mixtures favor the target annealed complex in solution, and the primer sequences exhibit minimal propensity toward homo/heterodimer formation due to the strict computational design. Experimental validation demonstrated the recovery of all 256 Gen_1 modular barcode sequences in parallel, emphasizing the degree of sample multiplexing provided by modular barcodes over other technologies, albeit with non-negligible off-target assembly events. The improved second-generation library was larger and had negligible off-target assembly outcomes. Further, the amplification of barcode DNA from mosquito-derived samples and in the context of contaminating human DNA was highly sensitive and specific. We used NGS to simultaneously read the barcodes for 256 Gen_1 samples, and separately, 96 Gen_2 samples. An analysis of NGS reads in our first-generation library allowed us to quantify polymerase error rates (deletions and substitutions). Observed error rates from the first-generation library motivated the design of a second-generation barcode library where amplicon errors would not prevent accurate assignment of the parent barcode. We confirmed that low complexity modular libraries did not increase the likelihood of substitution errors relative to previously reported values. Looking forward, modular DNA barcodes have potential for enhancing supply chain security and animal tracking by employing highly unique, microscopic markers capable of highly sensitive detection. Synthetic DNA in the environment remains vulnerable to degradation (e.g., by nucleases) [14]. Therefore, depending on the application, it may be helpful to boost the barcode half-life by embedding otherwise vulnerable DNA inside a protective matrix [26]. Barcode particles composed entirely of biomolecules are expected to be edible and biodegradable, resulting in broad application utility. 

## 4. Methods

### 4.1. Sequence Design

Flanking primer sequences for the barcodes were designed using Primer3 fed a random nucleotide sequence generated using an online random DNA sequence generator [27]. The block overhang regions and the terminal TrapTag primer sequence (Figure 1) were designed using NUPACK based on the target secondary structure [20]. For the Gen_1 library, variable region sequences were designed by first creating all possible 6 nt permutations from the 4 bases: A, T, C, G. The resulting list of 4096 sequences was filtered to remove homopolymers (e.g., sequences containing 4 or more identical, consecutive nucleotides) resulting in a list of 3936 sequences. From this list, groups of 4 randomly chosen sequences (performed 4000×) were used for the in silico testing of barcode assembly using NUPACK. The free energy of the target complex was recorded. The top 4 scoring complexes (e.g., complexes with the lowest free energy scores) were chosen for experimental validation. Notably, Hamming distance was not employed as an explicit design parameter for the Gen_1 library, but was rather calculated *after* sequence design, resulting in a value of 3. In contrast, for the Gen_2 library, we used custom Python code (DOI: 10.5281/zenodo.7415652) to identify a set of 24 variable region sequences for which all pairs met an explicit and more stringent Hamming dissimilarity cutoff. Specifically, the minimum Hamming distance was 7 between any two barcodes (and also a minimum Hamming distance of 5 even if an indel was to shift a barcode register ±1) (Figure S6, Table S3). All variant sequences are listed in Table 4. Following sequence design, all barcode variants were built in silico, and predicted assembly fidelity was assessed using NUPACK, ideally ensuring the target barcode remains the dominant complex at equilibrium despite possible off-target complex formation. Further code was written during Gen_2 library design for automating primer specificity checking against certain species or within the library itself (see section titled ‘Automated Primer Specificity Check’ in extended methods of SI).

### 4.2. Primer Specificity and Sensitivity

To further enhance the specificity of the chosen primers and to reduce chances of the off-target amplification of potentially contaminating DNA, each candidate primer pair designed using Primer3 was used for a BLAST search against multiple species that could contaminate barcode-positive samples. In addition, we considered downstream applications of this technology, which would require the amplification of small amounts of barcode DNA from complex sample types. In this case, we sought to design edible barcodes for marking mosquitoes [26]. Therefore, the species selected for the BLAST search were *Homo sapiens* and *Culicidae*. The BLAST procedure was automated using Python and the Biopython [28] NCBI command line functionality (custom Python scripts within “combinatorial_barcode_scripts.zip” hosted on Zenodo (DOI: 10.5281/zenodo.7415652). Following library construction in silico, primer specificity was again checked against the entire barcode library to ensure the no off-target mis-priming was likely to occur. Specifically, a FASTA file was created containing all sequences for the barcode library. Candidate Gen_2 TrapTag primer pairs were further evaluated in vitro for template specificity by attempting PCR-amplification of DNA extracted from *Culex tarsalis* and *Aedes aegypti* mosquitoes or human saliva, to verify the lack of off-target amplification of insect or contaminating human DNA (see SI, extended methods sections titled ‘in vitro Primer Sensitivity’ and ‘in vitro Primer Specificity’). The modular barcode library FASTA file was then converted to a custom database using Biopython. The BLAST procedure was repeated using the primer pair against the custom database to ensure that primers do not align internal to each barcode sequence.

### 4.3. Barcode Construction and Sequencing

Similar experimental validation and analysis methods were employed for both modular barcode libraries. For the Gen_1 library, the 32 oligos corresponding to the 4 variants for each of the 4 blocks were purchased from Integrated DNA Technologies (Coralville, Iowa) with 6 oligos containing a 5′ phosphate. Each oligo was resuspended to a stock concentration of 100 µM in duplex buffer (100 mM potassium acetate, 30 mM HEPES, pH 7.5). A 0.02 pmol/µL working solution was made from each stock solution using duplex buffer. From each of the 8 working solutions corresponding to a single modular barcode sequence, 2 µL was manually transferred to a 0.2 mL PCR tube and mixed. For the Gen_2 library, a liquid handling robot (OpenTrons OT-2) was employed for initial oligo mixing to reduce the chances of contamination. Oligo mixtures were heated to 94 °C for 4 min using a heat block, followed by gradual cooling for 1 hr by turning off the heat block. Following annealing, 2 µL T4 DNA Ligase Buffer (NEB) and 1µL T4 DNA Ligase (NEB) were added to the annealed mixture followed by incubation at room temperature for 10 min. The ligation reaction was heat inactivated by a 10-min incubation at 65 °C. Our OpenTrons Python pipetting script can be found within “combinatorial_barcode_scripts.zip” hosted on Zenodo (DOI: 10.5281/zenodo.7415652).

The inactivated ligation mixture product (102 bp for the Gen_1 library, 128 bp for the Gen_2 library) was used as the template for overhang PCR with the following reaction conditions: 1 cycle of 98 °C for 45 s, 30 cycles of 98 °C for 30 s, 61 °C for 30 s, 72 °C for 30 s, and 1 cycle of 72 °C for 1 min. Overhang PCR was performed using the Table 5 primer sets for amplifying barcode DNA, attaching the TrapTag and terminal sequencing adapters (Figure 1). All PCR reactions, including those for validating primer specificity, were performed using the same thermocycling conditions described immediately above for overhang PCR. Following amplification, PCR cleanup was performed using KAPA Pure Beads (Roche). Size selection for the 262 bp barcode library was performed using the Monarch DNA Gel Extraction Kit (New England Biolabs). The library was quantified using the Qubit 1X dsDNA HS Assay Kit (ThermoFisher), and each library member was diluted to 20 nM for sequencing sample prep. Paired-end 2 × 150 cycle sequencing was run on an Illumina NovaSEQ 6000 (Genomics and Microarray Core, University of Colorado Anschutz Medical Campus).

For NGS read analysis, the ea-utils package was used for initial sample processing, including adapter trimming and read joining. FastQC was used to check the overall quality of joined reads and to determine total read count of the detected barcode (Babraham Bioinformatics). Using the Biopython package and custom Python code, reads were aligned to a generic barcode template containing ‘N’s for the four variable sequence regions within each barcode (script can be found within “combinatorial_barcode_scripts.zip” hosted on Zenodo (DOI: 10.5281/zenodo.7415652)). Following alignment, demultiplexing was performed based on the TrapTag UMI appended to each individual positive sample. NGS data were analyzed by gathering various statistics on recovered reads, including, but not limited to, the number of reads containing no substitutions/deletions, the number/type of reads containing 1-nucleotide substitutions, and the number/type of reads containing variable nucleotide deletions. The average substitution rate per nucleotide was calculated for all non-indel reads by taking the average of the substitution rates determined for all nucleotide positions along the barcode, as described previously [25].

## Figures and Tables

**Table 1:** Modular barcode library parameters.

	Number of Blocks	Variants per Block	Number of Variable Nucleotides per Block	Minimum Hamming Distance between Variants	Total Number of Oligos Required	Total Number of Barcodes Allowed
Gen_1	4	4	6	3	32	256
Gen_2	3	8	12	7	48	512

**Table 2:** Designed Primer Sequences.

	Gen_1 Library		Gen_2 Library	
	Primer Sequence (5′–3′)	Design Source	Primer Sequence (5′–3′)	Design Source
Forward	CCAGTCCTCAACAAGCTG	Primer3	AGTGCGTGCAGTGAAAGC	Primer3
Reverse	GTTGAAGCCGGTTACCAC	Primer3	ATGGCGTTGCAAAGTCGG	Primer3
Trap Tag	TTCTGGGTTCCTCATCGC	Primer3	CGCCTTGATTTCAACTCGGCTCTCCGCTGAACA	NUPACK

**Table 3:** Barcode recovery by library for 1 M read subsets.

	Perfect Barcode Recovered (%)	Deletion Variants (%)	1-nt Substitution Variants (%)
Gen_1	80	3	5
Gen_2	26	1	3

**Table 4:** Modular barcode sequences designating each block variant.

Gen_1 Variable Region Sequences				Gen_2 Variable Region Sequences		
1	2	3	4	1	2	3
TGCGGC	GGCGTC	AGCGGG	CGCTCC	ATCGACTGCGAG	ATGACGAGTGCT	TGTCTCGAGTCT
				GCTAGCACTGAG	GAGATCTGCAGT	GCGCTGCTACTG
GCGGGC	CCCGGC	GCGGAA	ACTCGT	GTGTGCGCTAGC	CTATCGCGACGT	CACTCAGATGTG
				TGCTCTAGTAGC	CATGCTGTCAGC	GATGTGCAGAGA
TGGGCG	CCTACC	GCTGCC	ACGCGG	CGATACGAGATC	CGACGTCTATCG	TCTCGTCGTATG
				ACTGAGTGTCTC	GAGTCTACGTCG	CAGCAGTCTCGT
CGCCGG	GCACAG	GCGGGC	CCTTTG	TGCAGTGACTAG	GTCGCAGTACAG	CAGAGACAGCAG
				AGCGTGACGCGT	ACAGTGATCGAC	ATAGCGCACTCA

**Table 5:** Primer sequences used in overhang PCR for appending trap tag and sequencing adapters.

Primer Set #	Forward Primer (5′ – 3′)	Reverse Primer (5′ – 3′)
1	ACACTCTTTCCCTACACGACGCTCTTCCGATCTCCAGTCCTCAACAAGCTG	GTTGAAGCCGGTTACCAC
2	ACACTCTTTCCCTACACGACGCTCTTCCGATCT	TTCTGGGTTCCTCATCGCNNNNNNNNGTTGAAGCCGGTTACCAC
3	ACACTCTTTCCCTACACGACGCTCTTCCGATCT	GTGACTGGAGTTCAGACGTGTGCTCTTCCGATCTTTCTGGGTTCCTCATCGC
4	AATGATACGGCGACCACCGAGATCTACACTCTTTCCCTACACGACGCTCTTCCGATCT	CAAGCAGAAGACGGCATACGAGATNNNNNNNNNNATATTCACGTGACTGGAGTTCAGACGTGTGCTCTTCCGATCT

## Data Availability

The data and custom Python code presented in this study are openly available in Zenodo at DOI: 10.5281/zenodo.7415652.

## Supplementary Materials

The following supporting information can be downloaded at: https://www.mdpi.com/article/10.3390/ijms24032549/s1. Reference [29] is cited in the supplementary materials.
