# Supplementary material for: Scalable Combinatorial Assembly of Synthetic DNA for Tracking Applications
Source: Int J Mol Sci. 2023 Jan 29;24(3):2549. doi: 10.3390/ijms24032549 (PMC9917336; doi:10.3390/ijms24032549)
Supplement: Supplementary file 1 [file ijms-24-02549-s001.zip › ijms-2121067-supplementary.pdf]

Supplemental Information for
Scalable Combinatorial Assembly of Synthetic DNA for Tracking Applications

Julius D. Stuart [1], Natalie R. Wickenkamp [2], Kaleb A. Davis [2], Camden Meyer [3], Rebekah C. Kading [2] and Christopher D. Snow [1,3,*]

[1] Department of Chemistry, Colorado State University, Fort Collins, CO 80523, USA
[2] Department of Microbiology, Immunology, and Pathology, Colorado State University, Fort Collins, CO 80523, USA
[3] Department of Chemical and Biological Engineering, Colorado State University, Fort Collins, CO 80523, USA
* Correspondence: christopher.snow@colostate.edu; Tel.: +1-(970)-491-5276

Author Contributions: C.D.S., J.D.S. and R.C.K. designed the research; J.D.S., C.M., N.R.W. and K.A.D. performed the research; J.D.S. and C.M. analyzed the data; J.D.S. and C.D.S. wrote the paper; J.D.S., C.D.S. and R.C.K. edited the manuscript. All authors have read and agreed to the published version of the manuscript.

This PDF file includes:
Tables S1- 3
Figure S1-8
Extended Methods

Table S1. TrapTag Sequence Information

TrapTag_ oligo_ID	Illumina Index ID	TrapTag Index Sequence 5'-3' (8 nt)	TrapTag Oligo Sequence to order 5'-3' (51 nt) (contains *reverse complement* of TrapTag Index Sequence)
tt_001	UDI0001	CCGCGGTT	TTCTGGGTTCCTCATCGCAACCGCGGGTTGAA GCCGGTTACCAC
tt_002	UDI0002	TTATAACC	TTCTGGGTTCCTCATCGCGGTTATAAGTTGAA GCCGGTTACCAC
tt_003	UDI0003	GGACTTGG	TTCTGGGTTCCTCATCGCCCAAGTCCGTTGAA GCCGGTTACCAC
tt_004	UDI0004	AAGTCCAA	TTCTGGGTTCCTCATCGCTTGGACTTGTTGAA GCCGGTTACCAC
tt_005	UDI0005	ATCCACTG	TTCTGGGTTCCTCATCGCCAGTGGATGTTGAA GCCGGTTACCAC
tt_006	UDI0006	GCTTGTCA	TTCTGGGTTCCTCATCGCTGACAAGCGTTGAA GCCGGTTACCAC
tt_007	UDI0007	CAAGCTAG	TTCTGGGTTCCTCATCGCCTAGCTTGGTTGAA GCCGGTTACCAC
tt_008	UDI0008	TGGATCGA	TTCTGGGTTCCTCATCGCTCGATCCAGTTGAA GCCGGTTACCAC
tt_009	UDI0009	AGTTCAGG	TTCTGGGTTCCTCATCGCCCTGAACTGTTGAA GCCGGTTACCAC
tt_010	UDI0010	GACCTGAA	TTCTGGGTTCCTCATCGCTTCAGGTCGTTGAA GCCGGTTACCAC
tt_011	UDI0011	TCTCTACT	TTCTGGGTTCCTCATCGCAGTAGAGAGTTGAA GCCGGTTACCAC
tt_012	UDI0012	CTCTCGTC	TTCTGGGTTCCTCATCGCGACGAGAGGTTGA AGCCGGTTACCAC
tt_013	UDI0013	CCAAGTCT	TTCTGGGTTCCTCATCGCAGACTTGGGTTGAA GCCGGTTACCAC
tt_014	UDI0014	TTGGACTC	TTCTGGGTTCCTCATCGCGAGTCCAAGTTGAA GCCGGTTACCAC
tt_015	UDI0015V2	CAGTAGGC	TTCTGGGTTCCTCATCGCGCCTACTGGTTGAA GCCGGTTACCAC
tt_016	UDI0015	GGCTTAAG	TTCTGGGTTCCTCATCGCCTTAAGCCGTTGAA GCCGGTTACCAC
tt_017	UDI0016V2	TGACGAAT	TTCTGGGTTCCTCATCGCATTCGTCAGTTGAA GCCGGTTACCAC
tt_018	UDI0016	AATCCGGA	TTCTGGGTTCCTCATCGCTCCGGATTGTTGAA GCCGGTTACCAC
tt_019	UDI0017	TAATACAG	TTCTGGGTTCCTCATCGCCTGTATTAGTTGAA GCCGGTTACCAC
tt_020	UDI0018	CGGCGTGA	TTCTGGGTTCCTCATCGCTCACGCCGGTTGAA GCCGGTTACCAC
tt_021	UDI0019	ATGTAAGT	TTCTGGGTTCCTCATCGCACTTACATGTTGAA GCCGGTTACCAC

Table S1. TrapTag Sequence Information (Continued)

TrapTag_ oligo_ID	Illumina Index ID	TrapTag Index Sequence 5'-3' (8 nt)	TrapTag Oligo Sequence to order 5'-3' (51 nt) (contains *reverse complement* of TrapTag Index Sequence)
tt_022	UDI0020	GCACGGAC	TTCTGGGTTCCTCATCGCGTCCGTGCGTTGAA GCCGGTTACCAC
tt_023	UDI0021	GGTACCTT	TTCTGGGTTCCTCATCGCAAGGTACCGTTGAA GCCGGTTACCAC
tt_024	UDI0022	AACGTTCC	TTCTGGGTTCCTCATCGCGGAACGTTGTTGAA GCCGGTTACCAC
tt_025	UDI0023	GCAGAATT	TTCTGGGTTCCTCATCGCAATTCTGCGTTGAA GCCGGTTACCAC
tt_026	UDI0024	ATGAGGCC	TTCTGGGTTCCTCATCGCGGCCTCATGTTGAA GCCGGTTACCAC
tt_027	UDI0025	ACTAAGAT	TTCTGGGTTCCTCATCGCATCTTAGTGTTGAA GCCGGTTACCAC
tt_028	UDI0026	GTCGGAGC	TTCTGGGTTCCTCATCGCGCTCCGACGTTGAA GCCGGTTACCAC
tt_029	UDI0027	CTTGGTAT	TTCTGGGTTCCTCATCGCATACCAAGGTTGAA GCCGGTTACCAC
tt_030	UDI0028	TCCAACGC	TTCTGGGTTCCTCATCGCGCGTTGGAGTTGAA GCCGGTTACCAC
tt_031	UDI0029	CCGTGAAG	TTCTGGGTTCCTCATCGCCTTCACGGGTTGAA GCCGGTTACCAC
tt_032	UDI0030	TTACAGGA	TTCTGGGTTCCTCATCGCTCCTGTAAGTTGAA GCCGGTTACCAC
tt_033	UDI0031	GGCATTCT	TTCTGGGTTCCTCATCGCAGAATGCCGTTGAA GCCGGTTACCAC
tt_034	UDI0032	AATGCCTC	TTCTGGGTTCCTCATCGCGAGGCATTGTTGAA GCCGGTTACCAC
tt_035	UDI0033	TACCGAGG	TTCTGGGTTCCTCATCGCCCTCGGTAGTTGAA GCCGGTTACCAC
tt_036	UDI0034	CGTTAGAA	TTCTGGGTTCCTCATCGCTTCTAACGGTTGAA GCCGGTTACCAC
tt_037	UDI0035	AGCCTCAT	TTCTGGGTTCCTCATCGCATGAGGCTGTTGAA GCCGGTTACCAC
tt_038	UDI0036	GATTCTGC	TTCTGGGTTCCTCATCGCGCAGAATCGTTGAA GCCGGTTACCAC
tt_039	UDI0037	TCGTAGTG	TTCTGGGTTCCTCATCGCCACTACGAGTTGAA GCCGGTTACCAC
tt_040	UDI0038	CTACGACA	TTCTGGGTTCCTCATCGCTGTCGTAGGTTGAA GCCGGTTACCAC
tt_041	UDI0039	TAAGTGGT	TTCTGGGTTCCTCATCGCACCACTTAGTTGAA GCCGGTTACCAC
tt_042	UDI0040	CGGACAAC	TTCTGGGTTCCTCATCGCGTTGTCCGGTTGAA GCCGGTTACCAC

Table S1. TrapTag Sequence Information (Continued)

TrapTag_ oligo_ID	Illumina Index ID	TrapTag Index Sequence 5'-3' (8 nt)	TrapTag Oligo Sequence to order 5'-3' (51 nt) (contains *reverse complement* of TrapTag Index Sequence)
tt_043	UDI0041	ATATGGAT	TTCTGGGTTCCTCATCGCATCCATATGTTGAA GCCGGTTACCAC
tt_044	UDI0042	GCGCAAGC	TTCTGGGTTCCTCATCGCGCTTGCGCGTTGAA GCCGGTTACCAC
tt_045	UDI0043	AAGATACT	TTCTGGGTTCCTCATCGCAGTATCTTGTTGAA GCCGGTTACCAC
tt_046	UDI0044	GGAGCGTC	TTCTGGGTTCCTCATCGCGACGCTCCGTTGAA GCCGGTTACCAC
tt_047	UDI0045	ATGGCATG	TTCTGGGTTCCTCATCGCCATGCCATGTTGAA GCCGGTTACCAC
tt_048	UDI0046	GCAATGCA	TTCTGGGTTCCTCATCGCTGCATTGCGTTGAA GCCGGTTACCAC
tt_049	UDI0047	GTTCCAAT	TTCTGGGTTCCTCATCGCATTGGAACGTTGAA GCCGGTTACCAC
tt_050	UDI0048	ACCTTGGC	TTCTGGGTTCCTCATCGCGCCAAGGTGTTGAA GCCGGTTACCAC
tt_051	UDI0049	ATATCTCG	TTCTGGGTTCCTCATCGCCGAGATATGTTGAA GCCGGTTACCAC
tt_052	UDI0050	GCGCTCTA	TTCTGGGTTCCTCATCGCTAGAGCGCGTTGAA GCCGGTTACCAC
tt_053	UDI0051	AACAGGTT	TTCTGGGTTCCTCATCGCAACCTGTTGTTGAA GCCGGTTACCAC
tt_054	UDI0052	GGTGAACC	TTCTGGGTTCCTCATCGCGGTTCACCGTTGAA GCCGGTTACCAC
tt_055	UDI0053	CAACAATG	TTCTGGGTTCCTCATCGCCATTGTTGGTTGAA GCCGGTTACCAC
tt_056	UDI0054	TGGTGGCA	TTCTGGGTTCCTCATCGCTGCCACCAGTTGAA GCCGGTTACCAC
tt_057	UDI0055V2	GTTCGCCG	TTCTGGGTTCCTCATCGCCGGCGAACGTTGAA GCCGGTTACCAC
tt_058	UDI0055	AGGCAGAG	TTCTGGGTTCCTCATCGCCTCTGCCTGTTGAA GCCGGTTACCAC
tt_059	UDI0056V2	CACGAGCG	TTCTGGGTTCCTCATCGCCGCTCGTGGTTGAA GCCGGTTACCAC
tt_060	UDI0056	GAATGAGA	TTCTGGGTTCCTCATCGCTCTCATTCGTTGAAG CCGGTTACCAC
tt_061	UDI0057	TGCGGCGT	TTCTGGGTTCCTCATCGCACGCCGCAGTTGAA GCCGGTTACCAC
tt_062	UDI0058	CATAATAC	TTCTGGGTTCCTCATCGCGTATTATGGTTGAA GCCGGTTACCAC
tt_063	UDI0059	GATCTATC	TTCTGGGTTCCTCATCGCGATAGATCGTTGAA GCCGGTTACCAC
tt_064	UDI0060	AGCTCGCT	TTCTGGGTTCCTCATCGCAGCGAGCTGTTGAA GCCGGTTACCAC

Table S1. TrapTag Sequence Information (Continued)

TrapTag_ oligo_ID	Illumina Index ID	TrapTag Index Sequence 5'-3' (8 nt)	TrapTag Oligo Sequence to order 5'-3' (51 nt) (contains *reverse complement* of TrapTag Index Sequence)
tt_065	UDI0061	CGGAACTG	TTCTGGGTTCCTCATCGCCAGTTCCGGTTGAA GCCGGTTACCAC
tt_066	UDI0062	TAAGGTCA	TTCTGGGTTCCTCATCGCTGACCTTAGTTGAA GCCGGTTACCAC
tt_067	UDI0063	TTGCCTAG	TTCTGGGTTCCTCATCGCCTAGGCAAGTTGAA GCCGGTTACCAC
tt_068	UDI0064	CCATTCGA	TTCTGGGTTCCTCATCGCTCGAATGGGTTGAA GCCGGTTACCAC
tt_069	UDI0065	ACACTAAG	TTCTGGGTTCCTCATCGCCTTAGTGTGTTGAA GCCGGTTACCAC
tt_070	UDI0066	GTGTCGGA	TTCTGGGTTCCTCATCGCTCCGACACGTTGAA GCCGGTTACCAC
tt_071	UDI0067	TTCCTGTT	TTCTGGGTTCCTCATCGCAACAGGAAGTTGAA GCCGGTTACCAC
tt_072	UDI0068	CCTTCACC	TTCTGGGTTCCTCATCGCGGTGAAGGGTTGA AGCCGGTTACCAC
tt_073	UDI0069	GCCACAGG	TTCTGGGTTCCTCATCGCCCTGTGGCGTTGAA GCCGGTTACCAC
tt_074	UDI0070	ATTGTGAA	TTCTGGGTTCCTCATCGCTTCACAATGTTGAA GCCGGTTACCAC
tt_075	UDI0071	ACTCGTGT	TTCTGGGTTCCTCATCGCACACGAGTGTTGAA GCCGGTTACCAC
tt_076	UDI0072	GTCTACAC	TTCTGGGTTCCTCATCGCGTGTAGACGTTGAA GCCGGTTACCAC
tt_077	UDI0073	CAATTAAC	TTCTGGGTTCCTCATCGCGTTAATTGGTTGAA GCCGGTTACCAC
tt_078	UDI0074	TGGCCGGT	TTCTGGGTTCCTCATCGCACCGGCCAGTTGAA GCCGGTTACCAC
tt_079	UDI0075	AGTACTCC	TTCTGGGTTCCTCATCGCGGAGTACTGTTGAA GCCGGTTACCAC
tt_080	UDI0076	GACGTCTT	TTCTGGGTTCCTCATCGCAAGACGTCGTTGAA GCCGGTTACCAC
tt_081	UDI0077	TGCGAGAC	TTCTGGGTTCCTCATCGCGTCTCGCAGTTGAA GCCGGTTACCAC
tt_082	UDI0078	CATAGAGT	TTCTGGGTTCCTCATCGCACTCTATGGTTGAA GCCGGTTACCAC
tt_083	UDI0079	ACAGGCGC	TTCTGGGTTCCTCATCGCGCGCCTGTGTTGAA GCCGGTTACCAC
tt_084	UDI0080	GTGAATAT	TTCTGGGTTCCTCATCGCATATTCACGTTGAA GCCGGTTACCAC
tt_085	UDI0081	AACTGTAG	TTCTGGGTTCCTCATCGCCTACAGTTGTTGAA GCCGGTTACCAC
tt_086	UDI0082	GGTCACGA	TTCTGGGTTCCTCATCGCTCGTGACCGTTGAA GCCGGTTACCAC

Table S1. TrapTag Sequence Information (Continued)

TrapTag_ oligo_ID	Illumina Index ID	TrapTag Index Sequence 5'-3' (8 nt)	TrapTag Oligo Sequence to order 5'-3' (51 nt) (contains *reverse complement* of TrapTag Index Sequence)
tt_087	UDI0083	CTGCTTCC	TTCTGGGTTCCTCATCGCGGAAGCAGGTTGA AGCCGGTTACCAC
tt_088	UDI0084	TCATCCTT	TTCTGGGTTCCTCATCGCAAGGATGAGTTGAA GCCGGTTACCAC
tt_089	UDI0085	AGGTTATA	TTCTGGGTTCCTCATCGCTATAACCTGTTGAA GCCGGTTACCAC
tt_090	UDI0086	GAACCGCG	TTCTGGGTTCCTCATCGCCGCGGTTCGTTGAA GCCGGTTACCAC
tt_091	UDI0087	CTCACCAA	TTCTGGGTTCCTCATCGCTTGGTGAGGTTGAA GCCGGTTACCAC
tt_092	UDI0088	TCTGTTGG	TTCTGGGTTCCTCATCGCCCAACAGAGTTGAA GCCGGTTACCAC
tt_093	UDI0089	TATCGCAC	TTCTGGGTTCCTCATCGCGTGCGATAGTTGAA GCCGGTTACCAC
tt_094	UDI0090	CGCTATGT	TTCTGGGTTCCTCATCGCACATAGCGGTTGAA GCCGGTTACCAC
tt_095	UDI0091	GTATGTTC	TTCTGGGTTCCTCATCGCGAACATACGTTGAA GCCGGTTACCAC
tt_096	UDI0092	ACGCACCT	TTCTGGGTTCCTCATCGCAGGTGCGTGTTGAA GCCGGTTACCAC
tt_097	UDI0093	TACTCATA	TTCTGGGTTCCTCATCGCTATGAGTAGTTGAA GCCGGTTACCAC
tt_098	UDI0094	CGTCTGCG	TTCTGGGTTCCTCATCGCCGCAGACGGTTGAA GCCGGTTACCAC
tt_099	UDI0095	TCGATATC	TTCTGGGTTCCTCATCGCGATATCGAGTTGAA GCCGGTTACCAC
tt_100	UDI0096	CTAGCGCT	TTCTGGGTTCCTCATCGCAGCGCTAGGTTGAA GCCGGTTACCAC

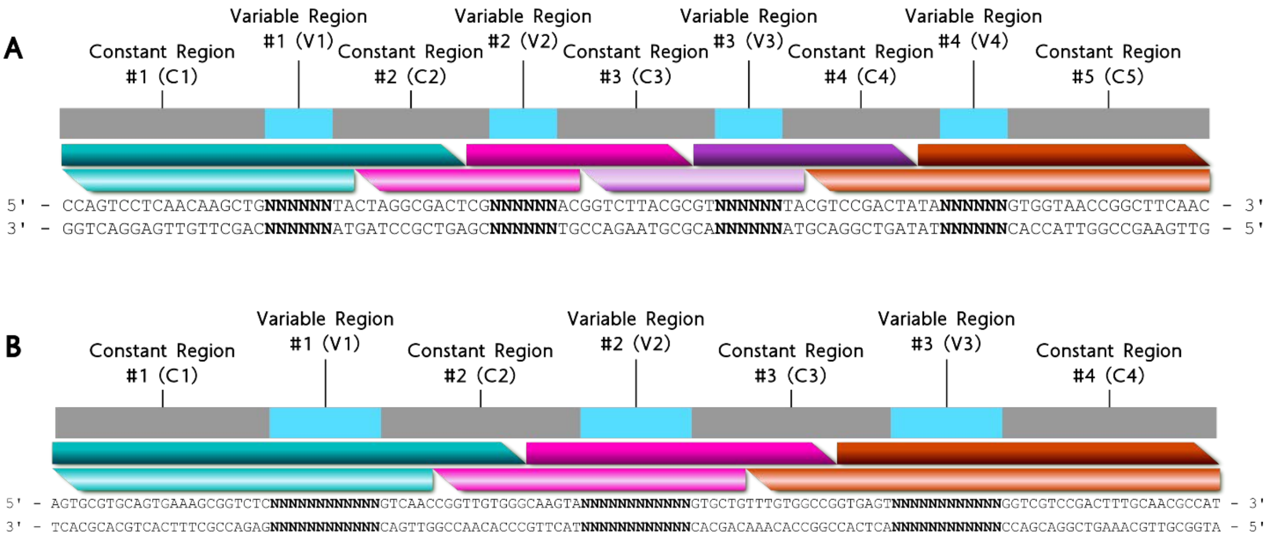

Figure S1. Modular Barcode Layout. A) Modular barcode layout for the Gen_1 library displaying 4 blocks containing a total of 4 variable region sequences. **B)** Modular barcode layout for the Gen_2 library displaying 3 blocks containing a total of 3 variable region sequences.

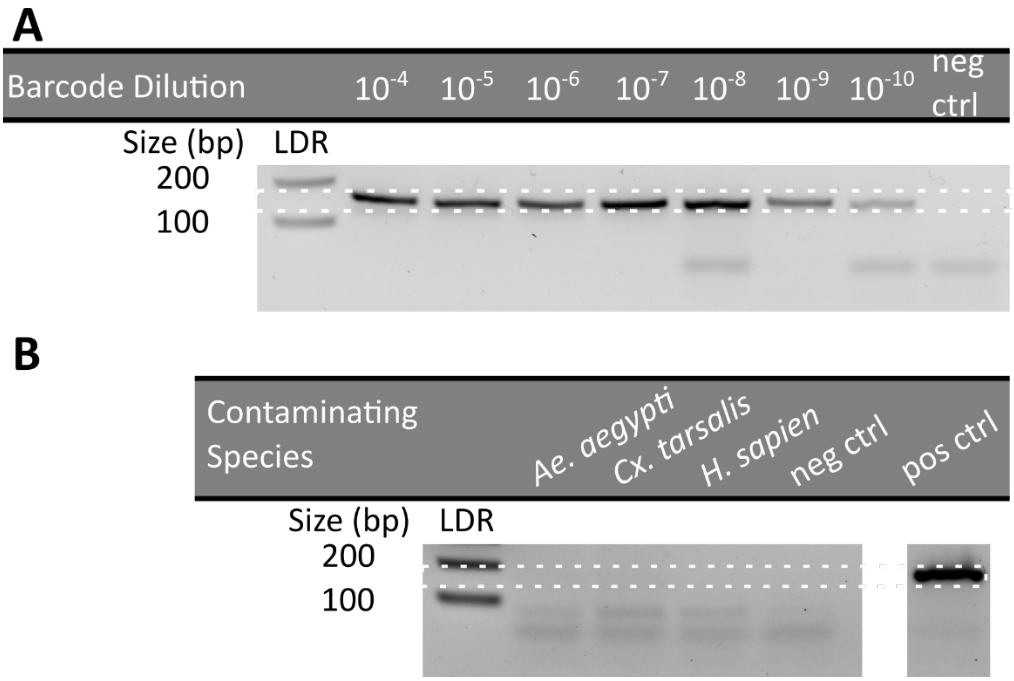

Figure S2. Primer Sensitivity and Specificity. A) Gel electrophoresis results following PCR with barcode template ranging from 10^{-4} – 10^{-10} dilutions of the initial barcode concentration, highlighting the sensitivity of the designed primer set for amplifying the target 161bp barcode amplicon. **B)** Gel electrophoresis results following PCR of barcode in the presence of additional contaminating species. *Ae. aegypti* refers to a pool of 15 *Aedes aegypti* mosquitos. *Cx. tarsalis* refers to a pool of 15 *Culex tarsalis* mosquitos. *H. sapien* refers to human saliva. Remarkably, the designed primer pair exhibits strict specificity for amplifying barcode only with no observed off-target amplification of contaminating species.

Problem: Identical 5' terminal nucleotides (highlighted below) allow off-target annealing

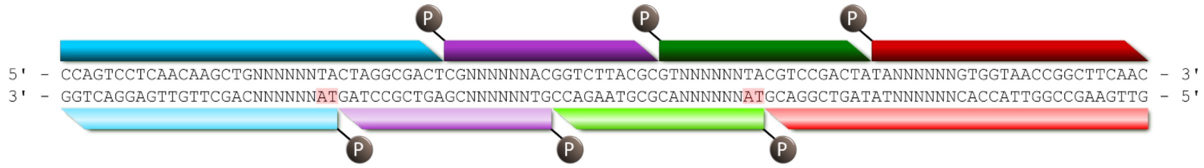

Solution:

1) Assign ***domain*** names to the 5' terminal end of the light blue and light green strands corresponding to the highlighted nucleotides..

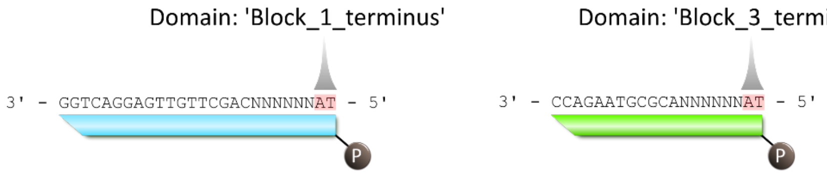

2) Use Nupack's ***Diversity*** constraint to force the specified domains to contain non-identical nucleotides during the design run.

my_hard_constraints = [. . .
Diversity(word = 4, types = 4, scope = [Block_1_terminus, Block_3_terminus])
. . .]

word: Length in nucletides of the 'window' probed by the constaint
types: The number of nucleotide types to include that must occur in the word
scope: accepts a list of ***concatenated*** domains for the constaint to act upon

Result: Following a design run, a unique nucleotide occupies each of the 4 positions in the 'Block_1_terminus' and 'Block_3_terminus' domains.

Figure S3. Negative Design with NUPACK. The 5’ terminal sequence regions of blocks 1 and 3, light blue and light green, respectively, are assigned domain names used as input for the Diversity constraint from NUPACK which prevents identical nucleotides from appearing in the specified domains following a design run.

Substitution Type	Frequency (%)	Frequency as reported by Pfeiffer et al. (%)
C -> T	0.010	0.11
G -> T	0.008	0.11
C -> A	0.006	0.13
G -> A	0.005	-
T -> G	0.004	-
A -> T	0.004	-
T -> C	0.003	0.04
A -> G	0.002	-
C -> G	0.002	0.04
A -> C	0.001	0.04
G -> C	0.001	0.04
T -> A	0.001	-

Table S2. Comparison of detected substitution frequencies with values reported by Pfeiffer *et al.*[1].

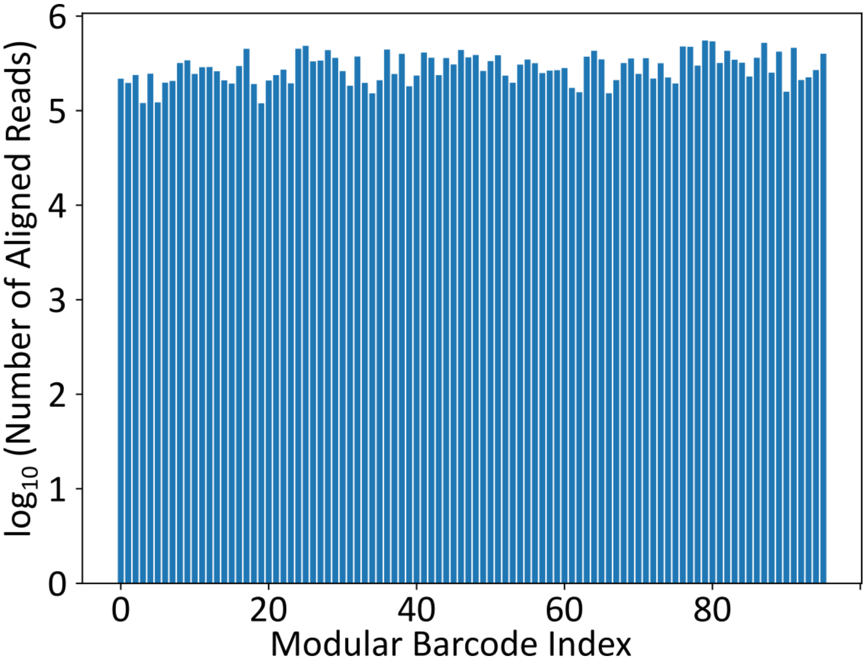

Figure S4. Gen_2 Barcode Recovery. Following alignment of ~109M joined reads using a custom Python script, all 96 pooled barcodes were detected at read quantities above 10^5.

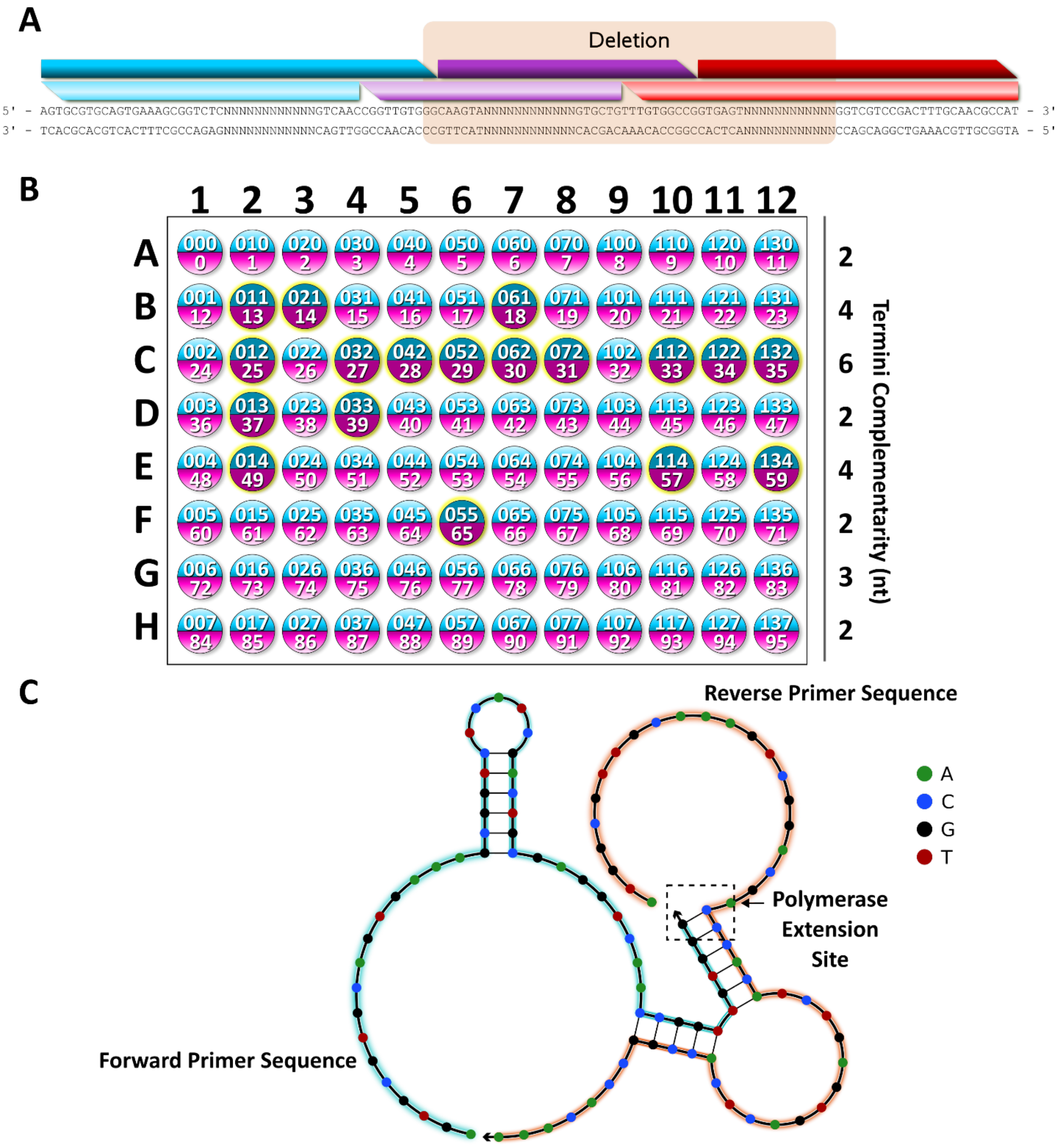

Figure S5. Gen_2 Deletion Variant Analysis. A) Flawed reads were very rare in the pooled 96 barcodes from the Gen_2 library (main text Fig. 6). Here we sought to better understand the origin of the fifth most common deletion variant (Fig. 6 row 5). The deletion spanned blocks 2 and 3 such that the only remaining variable region sequence originated in block 1. Of the 130 reads detected, 125 reads had 'intact' TrapTag sequences (e.g., no indels or substitutions), which were cross-referenced to the 96-well plate used for oligo mixing for discerning the origin of the off-target assembly. **B)** Layout of the 96 well plate configuration used for appending unique TrapTags to each of the 96 barcodes. For each well, the top 3 digits correspond to the "barcode", the variable region indices for each of the 3 blocks. The bottom digit corresponds to the TrapTag index UMI. Darker shaded wells indicate originating locations

for the off-target assembly shown in A. Thus, this rare off-target assembly nonetheless occurred in at least 18 distinct wells. Approximately 25% of all deletion variant reads resulted from well C4, containing block 1 variant index 0, block 2 variant index 3, and block 3 variant index 2 (i.e. barcode "032"). **C)** The predicted secondary structure of the block 1 variant index 0 top strand (cyan strand background) with the block 3 variant index 2 bottom strand (orange strand background) by NUPACK at the annealing temperature (58 °C) employed for barcode amplification. Notably, the six 3' terminal nucleotides for the block 1 strand (TGTGGG) have the potential to anneal to the last two bases in constant region 4 (CC) followed by the first four bases in the variable region sequence of the block 3 strand (CACA), allowing polymerase extension during PCR, resulting in formation of the detected deletion variant. This off-target 6-bp complementarity can occur between the third variant (index 2) for block 3 (which contains CACATCTGAGTG in the reverse strand) and any block 1 variant (which all end with TGTGGG) , which explains why the third row of the barcode assembly plate is the dominant source. In aggregate, 116 out of the 125 traceable reads (93%) originated in row C, corresponding to barcodes **2. The other two rows with the most unwanted block1 to block3 pairing correspond to block3 variant indices 1 and 4, which both feature a 4-bp complementary region since their variable regions start with CA in the reverse direction. The total size of the Watson-Crick complementarity region between the 3' terminus of forward block 1 and the reverse block 3 variants (that would lead to the observed deletion variant) is listed to the right of the corresponding row in the panel B table. Elimination of this off-target assembly may be a useful negative design principle for future combinatorial barcode library design.

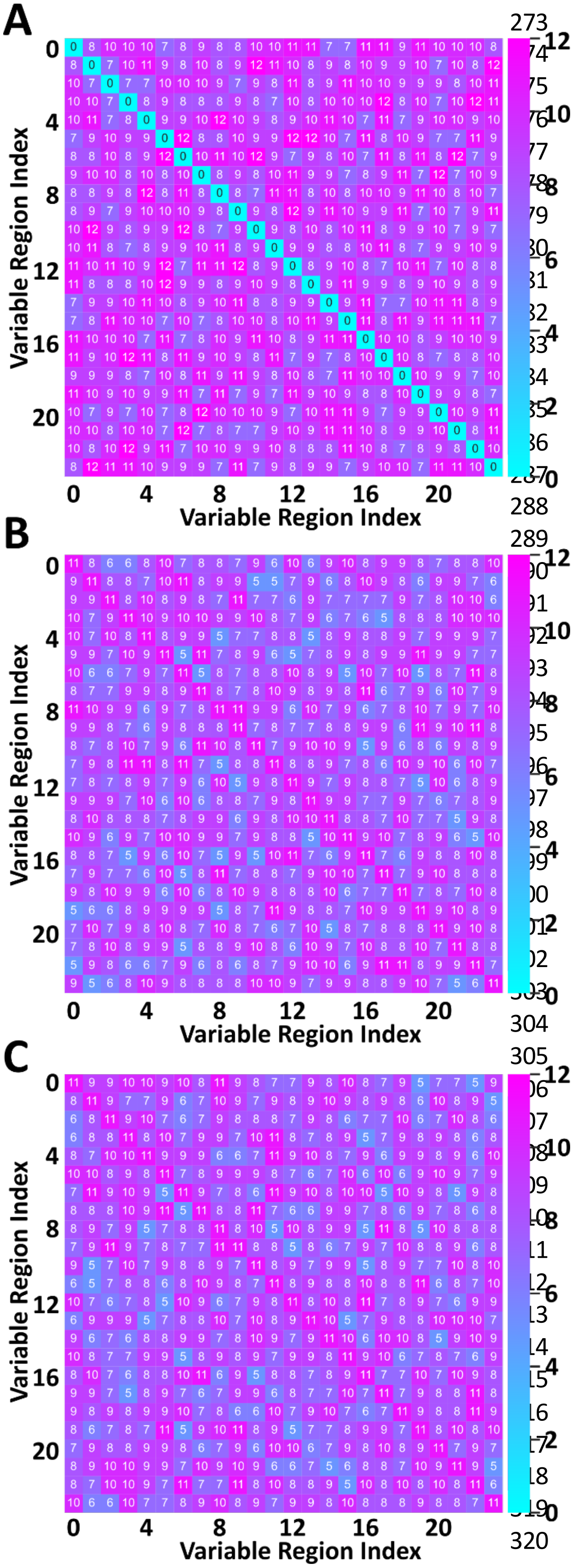

Figure S6. Variable Region Hamming Distance. (A) Heat map corresponding to the Hamming Distance results between all 24 designed variable region sequences. **(B, C)** Hamming distance results between all variable region sequences containing 1-nt insertions and 1-nt deletions, respectively.

Block	Block Variant	Variable Region Index	Variable Region Sequence (5' - 3'), top strand	Variable Region Sequence (5' - 3'), bottom strand
1	0	0	ATCGACTGCGAG	CTCGCAGTCGAT
	1	1	GCTAGCACTGAG	CTCAGTGCTAGC
	2	2	GTGTGCGCTAGC	GCTAGCGCACAC
	3	3	TGCTCTAGTAGC	GCTACTAGAGCA
	4	4	CGATACGAGATC	GATCTCGTATCG
	5	5	ACTGAGTGTCTC	GAGACACTCAGT
	6	6	TGCAGTGACTAG	CTAGTCACTGCA
	7	7	AGCGTGACGCGT	ACGCGTCACGCT
2	0	8	ATGACGAGTGCT	AGCACTCGTCAT
	1	9	GAGATCTGCAGT	ACTGCAGATCTC
	2	10	CTATCGCGACGT	ACGTCGCGATAG
	3	11	CATGCTGTCAGC	GCTGACAGCATG
	4	12	CGACGTCTATCG	CGATAGACGTCG
	5	13	GAGTCTACGTCG	CGACGTAGACTC
	6	14	GTCGCAGTACAG	CTGTACTGCGAC
	7	15	ACAGTGATCGAC	GTCGATCACTGT
3	0	16	TGTCTCGAGTCT	AGACTCGAGACA
	1	17	GCGCTGCTACTG	CAGTAGCAGCGC
	2	18	CACTCAGATGTG	CACATCTGAGTG
	3	19	GATGTGCAGAGA	TCTCTGCACATC
	4	20	TCTCGTCGTATG	CATACGACGAGA
	5	21	CAGCAGTCTCGT	ACGAGACTGCTG
	6	22	CAGAGACAGCAG	CTGCTGTCTCTG
	7	23	ATAGCGCACTCA	TGAGTGCGCTAT

Table S3. Assigned index values representing individual variable region sequences for Hamming distance analysis.

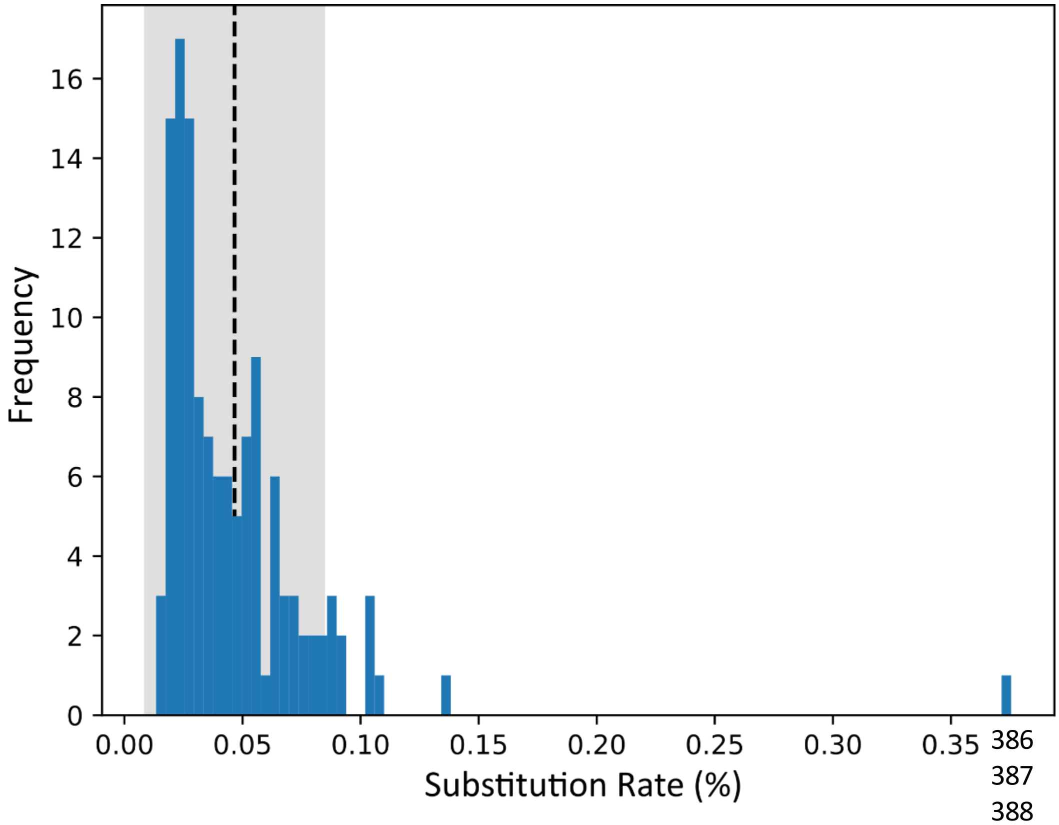

Figure S7. Histogram of 1-nt Substitutions. The dashed line represents the average substitution rate (0.05%). The gray shaded region represents ± the standard deviation (0.04%).

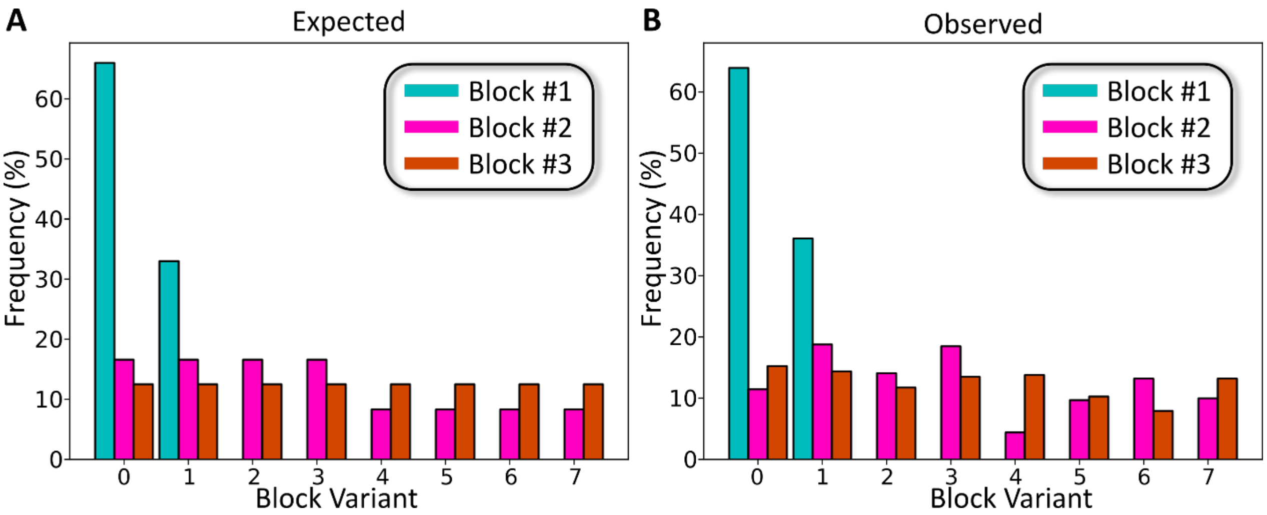

Figure S8. Here we assess the 341 pooled reads that constitute the most common deletion variant for the Gen_2 library (row 1 in main text Figure 6). **A)** Given the 96 barcode synthesis targets in play (Fig. S3B) we can compute the expected distribution of block variants for the pooled 96 barcodes assuming that an equimolar population of all barcodes was subjected to NGS and that the chance of a truncated NGS read does not depend on the barcode sequence. **B)** The observed distribution of block variants detected from the most common deletion variant for the Gen_2 library. The x-axis denotes the block variant and the y-axis denotes detection frequency as percent. The expected and observed frequencies are reasonably similar, consistent with the idea that the most common barcode read imperfection is simply a random endemic NGS artifact rather than a flaw in the barcode design and synthesis.

EXTENDED METHODS

Primer Design

Primer sequences for both Gen_1 and Gen_2 modular barcode libraries were designed using Primer3[2]. Specifically, a randomly generated 100,000nt sequence using an online sequence generator[3] was the input for the primer design code. Design parameters included primer sets with length of 18nt, melting temperature of 60 °C, GC content of 40 – 60% and a GC clamp of 2nt. The output designed 20 primer pairs were further sorted based on the sum of the following penalties assigned by Primer3: primer left self any TH, primer right self any TH, primer left self end TH, primer right self end TH, primer left hairpin TH, primer right hairpin TH, primer left end stability, primer right end stability, primer pair complementarity any TH, primer pair complementarity end TH. The primer pair with the lowest sum of penalties was chosen as barcode primer set.

Automated Primer Specificity Check

Custom Python code, available on Zenodo (DOI: 10.5281/zenodo.7415652), was written for checking for potential off-target amplification of candidate contaminating species (Culicidae, Homo sapiens). A spreadsheet of candidate primer pairs designed using Primer3 served as the input. After loading in the spreadsheet, the Biopython package is used for performing a blast search against the specified species for each primer pair. The program then parses the blast output files (xml) searching for instances where a primer pair align on opposing strands (requisite for exponential amplification) of an organism's genome and calculates the distance between that possible amplicon. Amplicon lengths much greater than the target barcode length (> 1kbp) were treated as non-threatening from a barcode detection/identification perspective. The results for each primer pair were written out to separate sub-directories for further analysis.

in vitro Primer Sensitivity

The barcode G-Block (5' – AGTGCGTGCAGTGAAAGCGGTCTCATCGACTGCGAGGTCAACCGGTTGTGGGC AAGTAATGACGAGTGCTGTGCTGTTTGTGGCCGGTGAGTTGTCTCGAGTCTGGTCGTCCGACTTTGCAACGCCAT – 3') was rehydrated to a 10ng/µL solution. 25µL of water was added to 250ng dried barcode. This solution was diluted 10^{-2} by adding 1µL of barcode solution to 99µL of water. This 10^{-2} solution was further diluted by adding 10µL of solution to 90µL of water and this serial dilution was repeated until the 10^{-10} dilution was achieved for each barcode. PCR was set up with primer set #1 (Table 5 of the main text) using the following master mix for each reaction. 25µL of GoTaq Green 2xMM, 2.5µL of 10µM forward primer, 2.5µL of 10µM reverse primer, 17µL of water, and 3µL of DNA template. 8 Reactions were prepared with the first 7 reactions using the previously prepared 10^{-4} through the 10^{-10} barcode as the template. The last remaining reaction used 3µL of water as the template for a negative control. Thermocycling of the two sets took place in tandem with the following conditions:

1. 96.0C° for 2:00min
2. 96.0C° for 20sec
3. 60.0C° for 20sec
4. 72.0C° for 30sec
5. Go to step 2 39 times
6. 72.0C° for 5:00min
7. 4.0C° for ∞

PCR product was run via gel electrophoresis on a 2% agarose gel in 1X TAE buffer at 90V for 60 minutes.

***in vitro* Primer Specificity**

The designed primers and barcode template were tested for specificity by running them against a panel of samples known to be negative for barcode. PCR was set up for primer set #1 (Table 5 of the main text) using the following master mix for each reaction: 25µL of GoTaq Green 2xMM, 2.5µL of 10µM NDX3-iseqFWD forward primer, 2.5µL of 10µM NDX3-Rev reverse primer, 17µL of water, and 3µL of DNA template. 9 reactions were prepared for each reaction set and the template samples for the first 8 were the same for each. Sample 1 a pool of 15 lab reared *Aedes aegypti* mosquitoes processed via the mosquito processing and extraction protocol. Sample 2 a pool of 15 lab reared *Culex tarsalis* mosquitoes processed via the mosquito processing and extraction protocol. Sample 3 was an aliquot of human saliva processed using the extraction protocol. Sample 4 water processed using the extraction protocol. Sample 5 a pool of 15 lab reared *Culex tarsalis* mosquitoes processed via the mosquito processing and extraction protocol. Sample 6 a pool of 15 lab reared *Aedes aegypti* mosquitoes processed via the mosquito processing and extraction protocol. Sample 7 was an aliquot of human saliva processed using the extraction protocol. Sample 8 water processed using the extraction protocol. Sample 9 was 10^{-9} diluted barcode.

Thermocycling of the two sets took place in tandem with the following conditions:

8. 96.0C° for 2:00min
9. 96.0C° for 20sec
10. 60.0C° for 20sec
11. 72.0C° for 30sec
12. Go to step 2 39 times
13. 72.0C° for 5:00min
14. 4.0C° for ∞

PCR product was run via gel electrophoresis on a 2% agarose gel in 1X TAE buffer at 90V for 60 minutes.

REFERENCES

1. Pfeiffer, F.; Gröber, C.; Blank, M.; Händler, K.; Beyer, M.; Schultze, J.L.; Mayer, G. Systematic evaluation of error rates and causes in short samples in next-generation sequencing. *Sci Rep-Uk* **2018**, *8*, 10950, doi:10.1038/s41598-018-29325-6.
2. Untergasser, A.; Cutcutache, I.; Koressaar, T.; Ye, J.; Faircloth, B.C.; Remm, M.; Rozen, S.G. Primer3--new capabilities and interfaces. *Nucleic Acids Res* **2012**, *40*, e115-e115, doi:10.1093/nar/gks596.
3. Stothard, P. The Sequence Manipulation Suite: JavaScript programs for analyzing and formatting protein and DNA sequences. *Biotechniques* **2000**, *28*, 1102-1104.
